# Is the Over-the-Head Technique an Alternative for Infant CPR Performed by a Single Rescuer? A Randomized Simulation Study with Lifeguards

**DOI:** 10.3390/pediatric16010010

**Published:** 2024-01-29

**Authors:** Silvia Aranda-García, Silvia San Román-Mata, Martín Otero-Agra, Antonio Rodríguez-Núñez, María Fernández-Méndez, Rubén Navarro-Patón, Roberto Barcala-Furelos

**Affiliations:** 1 GRAFAIS Research Group, Institut Nacional d’Educació Física de Catalunya (INEFC), Universitat de Barcelona (UB), 08038 Barcelona, Spain; silvia.aranda.garcia@gmail.com; 2 CLINURSID Research Group, Faculty of Nursing, University of Santiago de Compostela, 15782 A Coruña, Spain; antonio.rodriguez.nunez@sergas.es (A.R.-N.); mariajosefernandezmendez@gmail.com (M.F.-M.); 3 REMOSS Research Group, Faculty of Education and Sports Sciences, University of Vigo, 36005 Pontevedra, Spain; roberto.barcala@uvigo.es; 4 Nursing Department, University of Granada, 18071 Granada, Spain; 5 School of Nursing of Pontevedra, University of Vigo, 36001 Pontevedra, Spain; 6 Research Group in Simulation, Life Support and Intensive Care (SICRUS), Instituto de Investigación Sanitaria de Santiago de Compostela (IDIS), Santiago de Compostela, 15706 A Coruña, Spain; 7 Critical Pediatric Section, Pediatric Intermediate and Palliative Care, Hospital Clínico Universitario de Santiago, Santiago de Compostela, 15706 A Coruña, Spain; 8 RICORS of Primary Care Interventions to Prevent Maternal and Chronic Childhood Illnesses of Perinatal and Developmental Origin, RD21/0012/0025, Instituto de Salud Carlos III, 28220 Madrid, Spain; 9 Faculty of Teacher Training, University of Santiago de Compostela, 27001 Lugo, Spain; ruben.navarro.paton@usc.es

**Keywords:** infant CPR, one rescuer, over-the-head CPR, lifeguards

## Abstract

(1) Objective: The objective was to evaluate the quality of cardiopulmonary resuscitation (CPR, chest compressions and ventilations) when performed by a lone first responder on an infant victim via the over-the-head technique (OTH) with bag-mask ventilation in comparison with the standard lateral technique (LAT) position. (2) Methods: A randomized simulation crossover study in a baby manikin was conducted. A total of 28 first responders performed each of the techniques in two separate CPR tests (15:2 chest compressions:ventilations ratio), each lasting 5 min with a 15 min resting period. Quality CPR parameters were assessed using an app connected to the manikin. Those variables were related to chest compressions (CC: depth, rate, and correct CC point) and ventilation (number of effective ventilations). Additional variables included perceptions of the ease of execution of CPR. (3) Results: The median global CPR quality (integrated CC + V) was 82% with OTH and 79% with LAT (*p* = 0.94), whilst the CC quality was 88% with OTH and 80% with LAT (*p* = 0.67), and ventilation quality was 85% with OTH and 85% with LAT (*p* = 0.98). Correct chest release was significantly better with OTH (OTH: 92% vs. LAT: 62%, *p* < 0.001). There were no statistically significant differences in the remaining variables. Ease of execution perceptions favored the use of LAT over OTH. (4) Conclusions: Chest compressions and ventilations can be performed with similar quality in an infant manikin by lifeguards both with the standard recommended position (LAT) and the alternative OTH. This option could give some advantages in terms of optimal chest release between compressions. Our results should encourage the assessment of OTH in some selected cases and situations as when a lone rescuer is present and/or there are physical conditions that could impede the lateral rescue position.

## 1. Introduction

In infant CPR and in drowning cases, it is key to treat hypoxia as soon as possible because this is a potentially reversible cause of cardiac arrest [1,2,3]. According to the guidelines set forth by the European Resuscitation Council, it is recommended that the process of resuscitating infants commence with the administration of five initial rescue breaths before progressing to a combination of ventilation and chest compressions [3]. In optimal conditions, with the aim of improving ventilation in cardiac arrest cases in infants, a 15:2 ratio (chest compressions:breaths) is recommended when at least two trained first responders are on hand. One of them would position themselves to perform the ventilations, while the other would be responsible for administering chest compressions. Further, the use of pediatric bag-mask ventilation is recommended when performing ventilation [3]. In this setup, the first responder is advised to position themselves over the infant’s head (over-the-head: OTH), as this position allows for more effective ventilation from a technical perspective even when the procedures are carried out by healthcare experts [4]. Simultaneously, the task of administering chest compressions is undertaken by a second responder. This designated approach ensures a comprehensive and coordinated resuscitation strategy, maximizing the chances of successful outcomes during critical interventions.

However, the ideal conditions for administering resuscitation measures may not always be readily available or feasible, particularly in special circumstances where alterations in the underlying cause of the cardiac arrest, the chosen resuscitation approach, or the availability of essential resources may occur [5]. An example of this can be seen in infant drowning, where there is a possibility of a single trained rescuer being present (such as a lifeguard). Lifeguards, in this case, are often the only individuals available who have undergone comprehensive professional training and possess the necessary competence to administer immediate first-aid measures [6].

Likewise, over recent years, different variants of resuscitation techniques combining a lone responder performing ventilation movements and compressions whilst positioned at the head (OTH) have been examined in simulation models with adults [4,7]. In addition, a number of studies have been published that examine modified compression techniques in infants (<1 year old). However, these studies focus on finger placement in relation to the infant’s chest with two rescuers rather than the rescuer’s position in relation to the infant with a single rescuer [8,9,10,11]. Furthermore, other studies have compared the feasibility of OTH CPR with bag-mask ventilation versus pocket-mask ventilation in locations such as ambulances and hospitals [12,13]. The research efforts have yielded crucial insights into innovative methodologies and best practices targeted at improving the efficacy of resuscitation procedures for infants. These studies have extensively analyzed diverse approaches, exploring variations in chest compression techniques that involve the utilization of different finger placements and grips. The studies have also emphasized the importance of mitigating finger pain and fatigue during prolonged resuscitations [8]. Importantly, these investigations have maintained a steadfast focus on upholding the standard of resuscitative care, ensuring that the quality of resuscitation remains uncompromised. It is noteworthy that these studies focused on infant resuscitation performed by two rescuers. Despite this, a knowledge gap exists with regard to the way in which a lone first responder, who is trained to perform infant resuscitation, might be able to perform an alternative sequence with bag-mask ventilation, with the first responder standing over the infant’s head, and whether this approach could be optimal for a lone first responder trained to use bag-mask ventilation. In relation to the study of OTH-CPR in infants, to the best of our knowledge, this is the first research that discusses the topic of comparing two situations with the use of bag-mask ventilation and a single rescuer.

For this reason, under the hypothesis that a single first responder will perform high-quality cardiopulmonary resuscitation with a bag-mask while positioned over the head of the infant, the aim of the present study is to evaluate the quality of cardiopulmonary resuscitation (CPR, chest compressions and ventilations) when performed by a lone first responder on an infant victim via the over-the-head technique (OTH) with bag-mask ventilation in comparison with the standard lateral technique (LAT) position. 

## 2. Materials and Methods

### 2.1. Study Design

**Figure 1:**
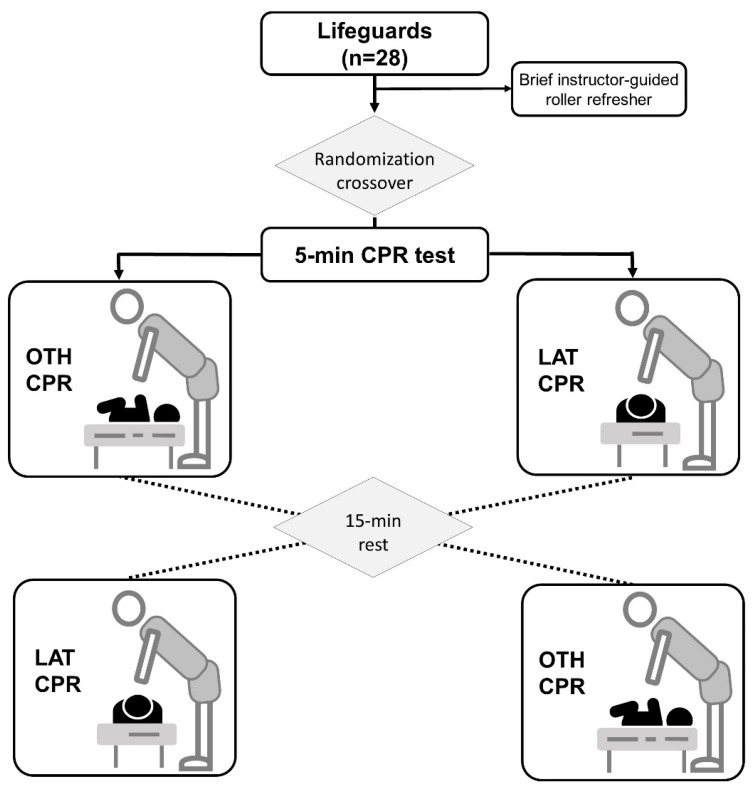
Flow-chart and design. CPR: cardiopulmonary resuscitation; OTH: over-the-head; LAT: lateral.

A randomized simulation crossover design was used to analyze differences between standard (LAT) and OTH CPR techniques during a 5 min CPR test. The order of the test was randomized, and there was a 15 min rest period between tests to minimize the effects of fatigue, as illustrated in Figure 1. This design facilitated a comprehensive and unbiased assessment of performance differences between standard CPR and OTH techniques. By implementing a randomized crossover approach, it was ensured that potential biases or order effects did not significantly influence the outcomes. Randomizing the order of the tests also helped counteract potential learning or adaptation effects to a specific technique, enabling a more accurate assessment of the intrinsic disparities between the two techniques. Furthermore, the 15 min rest period was a crucial measure to mitigate the effects of fatigue on the study participants. By ensuring that each test was conducted under a similar physical state, it was guaranteed that the observed differences were primarily attributable to variations in CPR techniques rather than accumulated fatigue. This consideration proved vital in drawing precise and dependable conclusions about the relative effectiveness of the evaluated CPR techniques.

### 2.2. Participants

The sample size was calculated based on an assumed minimum effect size (ES) of 0.5, an α error probability of 0.05, and a statistical power of 0.8. This produced a required sample size of 28 study participants as computed by G*Power 3.1.9.2 software (Heinrich—Heine—Universität, Düsseldorf, Germany). A final sample of 28 lifeguards was voluntarily recruited from students enrolled in the Sciences of Physical Activity and Sport degree delivered at the University of Vigo. In order to be included, participants had to meet the criteria of having previously been trained in the two infant resuscitation techniques under study, including training in ventilation with pediatric bag-mask ventilation. 

The final sample included in the present study had a median age of 21 years, with an interquartile range (IQR) of 20 to 23 years. Regarding the anthropometric characteristics, the median body weight was found to be 71 kg, with an IQR of 64 to 81 kg, and the median height was 1.75 m, with an IQR of 1.68 to 1.84 m. These detailed anthropometric measures were considered to account for the potential influence of participants’ physical constitution on the study outcomes. In terms of gender distribution, it was observed that a total of 32% of participants were female, with 9 female participants and 19 male participants. By including participants with a diverse yet representative range of weights and heights, a more accurate and comprehensive evaluation of the applicability of the findings to a broader range of individuals in the general population could be conducted.

Prior to their participation, all participants signed a written informed consent form, which clearly outlined the purposes, procedures, and potential risks associated with their involvement in the study. This process of obtaining informed consent was designed to ensure that all participants had a complete and clear understanding of the relevant details of the study, as well as their rights and options for participation. The protocol of this study underwent rigorous review and approval by the ‘blinded for the review process’ ethics committee. This approval was granted after a thorough assessment of the study’s methodology, as well as its ethical aspects and compliance with established ethical guidelines and regulations. The review by the ethics committee ensured that all aspects related to the integrity and wellbeing of the participants were duly considered and safeguarded in line with the strictest ethical and legal standards.

### 2.3. CPR Refresher for a Single Lifeguard

**Figure 2:**
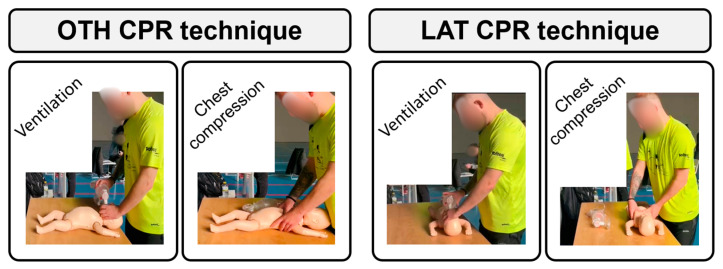
Both infant cardiopulmonary resuscitation (CPR) techniques using a single resuscitator: over-the-head (OTH) and standard (LAT).

Participants were trained in infant CPR during their training as lifeguards. Prior to starting the trials, each participant performed a brief instructor-guided roller refresher on CPR with a single lifeguard and pediatric bag-valve-mask ventilation (Ambu^®^ SPUR II, Denmark). This training was delivered with feedback via the QCPR training app (Laerdal, Norway) connected to the training manikin (Little Baby QCPR, Laerdal, Norway), which provides real-time information about the quality of resuscitation (chest compressions and ventilations). This equipment can be used to train individuals in both resuscitation techniques, which are mainly differentiated in the positioning of the first responder, in this case, a lifeguard, in relation to the infant: (1) standard CPR (LAT) with the first responder positioned to the side of the infant and (2) over-the-head CPR (OTH) (Figure 2) with them standing behind the infant and looking down over their head. None of the participants had actual experience performing in vivo infant CPR.

### 2.4. Infant CPR Techniques for a Single Lifeguard with Bag-Mask Ventilation

The action protocol for both techniques requires the performance of 5 initial ventilations, followed by 15 compressions and 2 ventilations, continuously, in accordance with European Resuscitation Council Guidelines (3). The ventilations were administered using a pediatric bag-mask ventilator, whilst chest compressions were executed employing the two-thumb encircling technique [8]. The infant manikin was laid out in the supine position on a firm table. 

In the case of the LAT CPR technique, which constitutes the control standard condition in the present study, the lifeguard stood next to the infant, in line with their trunk. In the OTH CPR technique, the lifeguard was positioned behind the infant, looking down over their head (“over-the-head”). In the case of this technique, chest compressions are also performed using the two-thumb encircling technique; however, in contrast to LAT, the hands are placed in the opposite direction (with the thumbs pointed down toward the feet of the infant as opposed to up toward their head) (Figure 2).

### 2.5. Assessment and Variables

An assessment of the 5 initial ventilations and 5 overall minutes of CPR following the standardized 15:2 compression-to-ventilation ratio was conducted meticulously using the QCPR training app (Laerdal, Norway). The app was connected to the training manikin, specifically the Little Baby QCPR (Laerdal, Norway), which accurately simulates real-life emergency scenarios to provide a realistic training experience. The data extracted from the software encompassed a comprehensive array of key performance indicators, which were meticulously analyzed to provide a comprehensive assessment of resuscitation quality. These indicators included the total number of cardiac compressions (in CC); the percentage of correct releases (in%), indicating the accuracy of the decompression phase; the percentage of chest compressions performed at the correct depth (in%); the mean chest compression depth (in mm), offering an average measure of the depth of compressions; the percentage of chest compressions performed at the correct rate (in%), the mean chest compression rate (in CC/min), providing an average measure of the rate of compressions per minute; the percentage of correct chest compression point (in%), reflecting the accuracy of the hand placement during compressions; ventilation (V) quality (in%), indicating the effectiveness of ventilation maneuvers; and the total count of effective ventilations delivered. Furthermore, to provide a comprehensive evaluation of the quality of cardiac chest compressions, the quality of cardiac chest compressions (CC quality, in%) was computed using a comprehensive formula that factored in the aspects of release, depth, rate, and correct CC point. This computation was crucial in obtaining a holistic assessment of the effectiveness and precision of the chest compression techniques employed during the simulated resuscitation efforts. The formula was as follows: (release + depth + rate + correct CC point)/4. Otherwise, an overall assessment of the resuscitation quality (CPR quality, in%) was determined by combining the CC quality and V quality using a weighted average. This approach allowed for a comprehensive and balanced evaluation of the participants’ overall performance, considering both the quality of chest compressions and the effectiveness of ventilations. It was calculated with the following formula: (CC quality + V quality)/2. These formulas have been examined in previous studies that also scrutinized the quality of cardiopulmonary resuscitation (7).

Moreover, following each test, participants were requested to provide feedback regarding their overall experience in performing CPR, including chest compressions and ventilations. Their subjective assessments of the ease of execution were meticulously analyzed utilizing a comprehensive 5-point Likert scale, where participants were prompted to rate their experience on a spectrum ranging from 0, representing “extremely easy”, to 5, signifying “extremely difficult”. This approach allowed for a nuanced understanding of the participants’ perceived challenges and comfort levels associated with the CPR techniques, facilitating a more comprehensive assessment of the practical aspects of CPR execution from the participants’ perspective.

### 2.6. Statistical Analysis

Statistical analysis was performed using the software IBM SPSS Statistics version 21 for Windows (Armonk, NY, USA). In the case of quantitative variables, firstly, the assumption of normality regarding data distribution was checked using the Shapiro–Wilk test. The median and interquartile range were used to characterize the data. The variables whose data followed a normal distribution were then compared as a function of the resuscitation technique using Student’s *t*-test for related samples. In the case of significant outcomes, effect sizes (ES) were calculated in accordance with Cohen’s d. With regards to variables whose data did not follow a normal distribution, variables were compared as a function of the resuscitation technique using the Wilcoxon rank sum test for related samples. In the case of significant outcomes, ES was calculated in accordance with Rosenthal’s r. ES was classified as follows: trivial (<0.2); small (0.2–0.5); moderate (0.5–0.8); large (0.8–1.3); and very large (≥1.3). Categorical variables were characterized using absolute and relative frequencies. Significance was set at *p* = 0.05 for all outcomes.

## 3. Results

**Figure 3:**
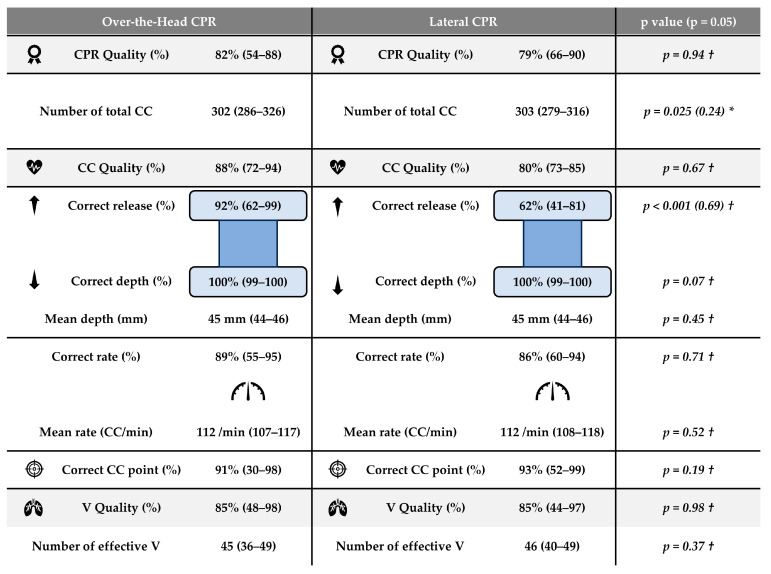
CPR variables (N = 28) for over-the-head (OTH) and control (LAT) conditions. CPR: cardiopulmonary resuscitation; CC: chest compressions; CC/min: chest compressions by minute; V: ventilation. * Variables described by mean ± standard deviation (SD) and 95% confidence interval. Comparisons by Student’s *t* test for related measures and Effect Size (in brackets) by Cohen’s d test. † Variables described by median and interquartile range. Comparisons by Wilcoxon’s signed ranked test for related measures and Effect Size (in brackets) by Rosenthal’s r test.

The results obtained from the cardiopulmonary resuscitation (CPR) test variables are graphically represented in Figure 3. CPR quality of greater than 70% was achieved using both resuscitation techniques with no statistically significant differences emerging between the two examined conditions (OTH: 82%; IQR 54–88% vs. LAT: 79%; IQR 66–90%, *p* = 0.94). CC quality also showed that values above 70% were achieved using both resuscitation techniques with no significant differences between the two conditions examined (OTH: 88%; IQR 72–94%; vs. LAT: 80%; IQR 73–85%, *p* = 0.67). Values above 70% were also found for the variable V quality, with no differences between the two techniques (OTH: 85%; IQR 48–98% vs. LAT: 85%; IQR 44–97%, *p* = 0.98). The only variables where significant differences were found were correct release, where OTH had higher values than LAT (OTH: 92%; IQR 62–99% vs. LAT: 62%; IQR 41–81%, *p* < 0.001), and the number of total CC, where OTH had lower values than LAT (OTH: 302 CC; IQR 286–326 CC vs. LAT: 303 CC; IQR 279–316%, *p* = 0.025). Furthermore, the other resuscitation variables studied showed no significant differences between the two techniques examined: correct depth (OTH: 100%; IQR 99–100% vs. LAT: 100%; IQR 99–100%, *p* = 0.07); mean depth (OTH: 45 mm; IQR 44–46 mm vs. LAT: 45 mm; IQR 44–46 mm, *p* = 0.45); correct rate (OTH: 89%; IQR 55–95% vs. LAT: 86%; IQR 60–94%, *p* = 0.71); mean rate (OTH: 112 CC/min; IQR 107–117 CC/min vs. LAT: 112 CC/min; IQR 108–118 CC/min, *p* = 0.52); correct CC point (OTH: 91%; IQR 30–98% vs. LAT: 93%; IQR 52–99%, *p* = 0.19); and number of effective V (OTH: 45 V; IQR 36–49 V vs. LAT: 46 V; IQR 40–49 V, *p* = 0.37).

The results derived from the ratings concerning participants’ perceptions of the ease of execution are comprehensively displayed in Table 1. Participants indicated a pronounced preference for the OTH technique over the LAT technique in terms of perceived ease of performing CPR, as evidenced by statistical data. The participants’ perceptions revealed that executing CPR using the OTH technique was significantly more manageable, with a median score of 2 (interquartile range (IQR): 2–3) in contrast to a median score of 3 (IQR: 3–3) for the LAT technique, with a *p*-value of 0.004. Moreover, participants also reported that both CC and V were notably easier to execute when employing the OTH approach compared to the LAT technique. Specifically, the perceived ease of performing CC was rated as 2 (IQR: 2–3) for the OTH technique in contrast to a rating of 3 (IQR: 2–4) for the LAT technique, with a *p*-value of 0.024. Similarly, participants found it more manageable to perform ventilations using the OTH technique, with a perceived ease score of 2 (IQR: 1–3) compared to a score of 3 (IQR: 2–3) for the LAT technique, with a *p*-value of 0.004.

## 4. Discussion

The standard position for performing cardiopulmonary resuscitation in infants is based on consensus recommendations, as comprehensive tests regarding alternative positions have not been conducted thus far. This creates a significant knowledge gap, particularly in situations where the standard position cannot be assumed due to specific circumstances. Considering the lack of extensive research on alternative positions for infant CPR, the study aimed to evaluate the quality of CPR performed by a lone first responder (lifeguard) on an infant victim using the OTH technique in comparison with the more typically performed LAT technique. The main findings are that the OTH technique is feasible and at least noninferior to the standard technique. In an infant manikin in controlled conditions, good resuscitation quality was achieved both overall and in relation to bag-mask ventilations and chest compressions. Additionally, OTH seemed to allow more efficient chest recoil following chest compressions (CC). An examination of the present findings suggests that this technique could be an effective alternative in instances in which only a single first responder trained in handling bag-mask ventilation is present, for instance, as is often the case with swimming pool lifeguards.

This finding may be important given that current European Resuscitation Council guidelines around the pediatric action protocol stipulate that bag-mask ventilation be used when at least two trained rescuers perform resuscitation [3]. Lifeguards, who are paramedics with the duty to assist, have the capacity to learn different CPR techniques adapted to the reality of their professional practice [7,14]. It is essential that they incorporate non-conventional situations and positions into their training in order to achieve optimal quality standards when facing adverse situations. The significance of our findings lies herein. On the one hand, it provides evidence of the quality of resuscitation that a lifeguard can achieve when performing resuscitation alone using both techniques (standard LAT and OTH) and employing a bag-mask for ventilation. On the other hand, it introduces a new rescuer position that enables an alternative technique (OTH) equally as effective as the conventional one. The lifeguard’s ultimate goal is to deliver high-quality CPR (including chest compressions and ventilations) regardless of the technique used, as achieved by our participants with CPR qualities exceeding the desired 70% [15].

In the context of infant cardiopulmonary resuscitation, ventilation is considered crucial because it can reverse cardiac arrest [3]. In the present study, participants consistently achieved 85% ventilation quality using bag-mask ventilation, regardless of technique. This high efficacy mirrors outcomes achievable when two rescuers collaborate in infant CPR using different techniques (means Q–V: between 82% and 86%) [8]. The current findings exceed the results of previous studies on the OTH technique with adults [4,7]. In the pioneering study, paramedics achieved only 17% effective ventilations [4]. Further, another study on OTH CPR involving two rescuers demonstrated slightly lower-quality ventilations (median of 71%) [7] compared to the outcomes of this research, possibly because participants lacked strong airway skills [4] or performed resuscitation on a moving boat at 20 knots [7]. In line with positive ventilation outcomes, both techniques showed good-quality CC. The OTH technique demonstrated better recoil compared to the standard LAT technique (92% OTH vs. 62% LAT, *p* < 0.001), consistent with findings from the earlier pioneering study [4]. The difference in recoil performance may be due to how rescuers position themselves during compression, affecting their center of gravity and potentially improving chest recoil efficiency. This highlights the importance of considering rescuers’ biomechanics to optimize resuscitation and emphasizes the potential of the OTH technique in enhancing overall effectiveness.

In accordance with participant feedback, the novel “over-the-head” (OTH) infant resuscitation technique, designed for lone rescuers, was perceived as more straightforward to execute when compared to the standard resuscitation method. This applies equally to chest compressions (CC) as well as ventilations. Lifeguards or healthcare professionals are likely familiar with the OTH position as it is recommended for infant ventilation in some techniques involving at least two rescuers (e.g., two-thumb encircling or two-finger) [8]. Our findings suggest that this technique may be beneficial for both ventilations and CC, enabling a single rescuer to perform complete CPR in individual care scenarios. A lifeguard must learn various techniques for their professional praxis and adhere to recommendations, selecting the best technique for the victim based on their own training and skill. Based on the present findings, it may be useful to include the novel OTH technique in training programs and CPR recommendations, especially when similar scenarios to those examined in the present study arise, in which a lone responder must attend to an infant.

This work presents limitations that should be pointed out. Firstly, it is a study carried out with a small and local sample of trained lifeguards. While it yielded interesting preliminary findings, conducting the same tests with a major sample and greater diversity regarding participant backgrounds could lead to different results (for instance, the sample displays limited diversity concerning age). And second, it was performed in a controlled simulation scenario and with a manikin. With real victims, the difficulty of resuscitation will be different and more complex.

It is important to highlight the practical usefulness of reported outcomes, whose application in the field could be valuable and provide a number of perspectives for future research. With regards to the infant OTH resuscitation technique used in the present study, good handling concerning ventilation movements was expected because the positioning of the rescuer is the same as when resuscitation is performed through the collaboration of two individuals, with this positioning, specifically, simplifying the technique. Nonetheless, the overall quality of resuscitation or of chest compressions could not have been predicted to be as good as was found here. Despite being a different technique in which the rescuer spends the whole time at the head of the infant looking down (“over-the-head”) and makes a different kind of contact during chest compressions, the present outcomes produce evidence that OTH CPR is a good alternative option when a lone rescuer is charged with ventilating an infant.

Promising areas are identified for future research that may broaden understanding and provide more in-depth knowledge in the field of resuscitation with a lone responder and bag-mask ventilation. It would be desirable that future research include more participants and cover different settings whilst maintaining a high skill level executing the technique in order to obtain a more representative perspective. Possible future research directions include the evaluation of other resuscitation techniques with a lone responder and bag-mask ventilation, analysis of the effectiveness of this resuscitation approach within different sociodemographic groups, and examination of this technique in comparison with other resuscitation methods, such as the mouth-to-mouth ventilation technique and use of a pocket mask.

## 5. Conclusions

Chest compressions and ventilations can be performed with similar quality in an infant manikin by a lone lifeguard both with the standard recommended position (LAT) and the alternative OTH. This option could give some advantages in terms of optimal chest release between compressions. Our results should encourage the assessment of OTH in some selected cases and situations as when a lone rescuer is present and/or there are physical conditions that could impede the lateral rescue position. Future studies are needed to have larger and more diverse samples, as well as comparisons of this technique with other ventilation methods in resuscitation situations carried out by a single lifeguard.

## Figures and Tables

**Table 1:** Perceptions of the ease of executing the two resuscitation techniques (N = 28).

	Over-the-Head CPR		Lateral CPR		*p*-Value (*p* = 0.05)
Ease of CPR	2	(2–3)	3	(3–3)	*p* = 0.004 (0.55) †
Ease of CC	2	(2–3)	3	(2–4)	*p* = 0.024 (0.43) †
Ease of V	2	(1–3)	3	(2–3)	*p* = 0.004 (0.54) †

CPR: cardiopulmonary resuscitation; CC: chest compressions; V: ventilation. † Variables characterized according to median and interquartile range. Significance testing conducted via Wilcoxon’s signed rank test for related samples, and effect size (in brackets) calculated via Rosenthal’s r test. Effect size classification: <0.2 trivial; (0.2–0.5) small; (0.5–0.8) moderate; (0.8–1.3) large; ≥1.3 very large. CPR: cardiopulmonary resuscitation; CC: chest compressions; V: ventilation.

## Data Availability

The data presented in this study are available on request from the corresponding author.

## References

[1] Manglick M.P., Ross F.I., Waugh M.-C., A Holland A.J., Cass D.T., Soundappan S.S.V. (2018). Neurocognitive outcomes in children following immersion: A long-term study. Arch. Dis. Child..

[2] Truhlář A., Deakin C.D., Soar J., Khalifa G.E.A., Alfonzo A., Bierens J.J.L.M., Brattebø G., Brugger H., Dunning J., Hunyadi-Antičević S. (2015). European Resuscitation Council Guidelines for Resuscitation 2015: Section 4. Cardiac arrest in special circumstances. Resuscitation.

[3] Van de Voorde P., Turner N.M., Djakow J., de Lucas N., Martinez-Mejias A., Biarent D., Bingham R., Brissaud O., Hoffmann F., Johannesdottir G.B. (2021). European Resuscitation Council Guidelines 2021: Paediatric Life Support. Resuscitation.

[4] Ćwiertnia M., Kawecki M., Ilczak T., Mikulska M., Dutka M., Bobiński R. (2019). Comparison of standard and over-the-head method of chest compressions during cardiopulmonary resuscitation—A simulation study. BMC Emerg. Med..

[5] Lott C., Truhlář A., Alfonzo A., Barelli A., González-Salvado V., Hinkelbein J., Nolan J.P., Paal P., Perkins G.D., Thies K.-C. (2021). European Resuscitation Council Guidelines 2021: Cardiac arrest in special circumstances. Resuscitation.

[6] Koon W., Schmidt A., Queiroga A.C., Sempsrott J., Szpilman D., Webber J., Brander R. (2020). Need for consistent beach lifeguard data collection: Results from an international survey. Inj. Prev..

[7] Barcala-Furelos R., Carracedo-Rodríguez E., Lorenzo-Martínez M., Alonso-Calvete A., Otero-Agra M., Jorge-Soto C. (2023). Assessment of over-the-head resuscitation method in an inflatable rescue boat sailing at full speed. A non-inferiority pilot study. Am. J. Emerg. Med..

[8] Barcala-Furelos R., Barcala-Furelos M., Cano-Noguera F., Otero-Agra M., Alonso-Calvete A., Martínez-Isasi S., Aranda-García S., López-García S., Rodríguez-Núñez A. (2022). A Compar-ison between Three Different Techniques Considering Quality Skills, Fatigue and Hand Pain during a Prolonged Infant Resuscita-tion: A Cross-Over Study with Lifeguards. Children.

[9] Smereka J., Kasiński M., Smereka A., Ładny J.R., Szarpak Ł. (2017). The quality of a newly developed infant chest compression method applied by paramedics: A randomised crossover manikin trial. Kardiol. Pol..

[10] Smereka J., Szarpak L., Smereka A., Leung S., Ruetzler K. (2017). Evaluation of new two-thumb chest compression technique for infant CPR performed by novice physicians. A randomized, crossover, manikin trial. Am. J. Emerg. Med..

[11] Szarpak L., Smereka J., Ladny J.R., Ruetzler K. (2019). The thumbs angle used in the novel infant chest compression technique (new two-thumb technique, nTTT) can influence the quality parameters of resuscitation. Med. Intensiv..

[12] Jo C.H., Cho G.C., Lee C.H. (2017). Two-thumb encircling technique over the head of patients in the setting of lone rescuer infant CPR occurred during ambulance transfer: A crossover simulation study. Pediatr. Emerg. Care.

[13] Jo C.H., Jung H.S., Cho G.C., Oh Y.J. (2015). Over-the-head two-thumb encircling technique as an alternative to the two-finger tech-nique in the in-hospital infant cardiac arrest setting: A randomised crossover simulation study. Emerg. Med. J..

[14] Adelborg K., Dalgas C., Grove E.L., Jørgensen C., Al-Mashhadi R.H., Løfgren B. (2011). Mouth-to-mouth ventilation is superior to mouth-to-pocket mask and bag-valve-mask ventilation during lifeguard CPR: A randomized study. Resuscitation.

[15] Perkins G.D. (2007). Simulation in resuscitation training. Resuscitation.

